# Germ Cell Isolation and Cryopreservation from Reproductive Organs of Brown Mealworm

**DOI:** 10.3390/insects13121108

**Published:** 2022-11-30

**Authors:** Do Gyeung Byeun, Byoung-San Moon, Seungki Lee, Jung Kyu Choi

**Affiliations:** 1 Department of Biotechnology, College of Life and Applied Sciences, Yeungnam University, Gyeongsan 38541, Republic of Korea; 2 Department of Biotechnology, Chonnam National University, Yeosu 59626, Republic of Korea; 3 Biological and Genetic Resources Assessment Division, National Institute of Biological Resources, Incheon 404-708, Republic of Korea

**Keywords:** brown mealworm, vitrification, Styrofoam, germ cells, cryopreservation, fertility, Tenebrio

## Abstract

**Simple Summary:**

Long-term breeding of mealworms leads to in inbreeding weakness, such as decreased egg production, decreased survival rate, and increased growth period. In this study, we isolated and freezed germ cells from the superior brown mealworm. Male and female adult brown mealworms were distinguished according to the presence or absence of protrusion at the tip of the five segments from the abdomen. We observed sperm and ovarioles in anatomically isolated testes and ovaries. The mechanical and enzymatic (collagenase I) methods were used to isolate ovarioles from adult female brown mealworms. In the enzymatic method, most were torn and burst as the membrane of the ovarioles was damaged by collagenase I, unlike the mechanical method. We also cryopreserved the ovaries of female brown mealworms using slow freezing and vitrification. We found that the yolk sac was completely damaged in the ovaries after slow freezing. However, only partial damage was achieved in the vitrification group compared to the control group (no freezing). The newly developed vitrification method with alginate-encapsulated ovarioles maintained the yolk sac in the ovarioles but was evenly distributed. This study provides basic data for reproductive studies of other useful insects.

**Abstract:**

This study aimed to isolate and freeze germ cells from the superior brown mealworm. Styrofoam diet changes were observed for 20 days to determine whether mealworms were useful insects for decomposing Styrofoam. The average weight of mealworms before the Styrofoam diet was 500 mg, which decreased to 336 mg at D20 after their diet. To preserve mealworms with excellent Styrofoam-degrading ability, we first isolated the reproductive organs of mealworms, testes, ovaries, sperms, and ovarioles. Morphologically, male and female adult brown mealworms were distinguished according to the presence or absence of a protrusion at the tip of the fifth segment of the abdomen. Sperms and ovarioles were observed in anatomically isolated testes and ovaries. We compared mechanical and enzymatic (collagenase I) methods to effectively isolate ovarioles from adult female brown mealworms. For the enzymatic method, most were torn and burst as the membrane of the ovarioles was damaged by collagenase I, unlike the mechanical method. To preserve the superior genetic resources of mealworms, we cryopreserved the ovaries of female brown mealworms using slow-freezing and vitrification. Histological analysis showed that the yolk sac was completely damaged in the ovaries after slow-freezing. However, only partial damage was achieved in the vitrification group compared to the control group (no freezing). The newly developed vitrification method with alginate-encapsulated ovarioles maintained the yolk sac in the ovarioles but was evenly distributed. These results provide basic data for reproductive studies of other useful insects and contribute to the biobanking and fertility preservation of superior mealworm germ cells and endangered insects.

## 1. Introduction

The brown mealworm is a genus of grain mealworms in the Coleoptera family, and its scientific name is *Tenebrio molitor*. It inhabits China, Japan, and South Korea, and the larvae of stored grain pests are called mealworms [1]. It is used to solve the global food supply and demand problem and as food for companion animals. Thus, it is attracting attention as a new protein-supplying food. The nutritional component of the mealworm, a brown mealworm larva, is 50.32% protein, which is much higher than in pigs (about 20% protein) and cattle (about 17% protein) [2]. In addition to food, there has also been a lot of interest in the environmental field. The intestinal microbes of mealworms decompose plastics that cause environmental problems; they are excreted in biodegradable feces [3,4,5]. For the industrial application of mealworms, it is necessary to establish large-scale breeding systems. However, long-term breeding results in inbreeding weakness due to inbreeding and degradation, such as decreased egg production, decreased survival rate, and an increased growth period [6,7]. To address these problems, it is necessary to secure mealworms with excellent genetic traits and to cryopreserve germ cells. However, there have been no studies on germ cells and freezing in insects. *Tenebrio molitor* is a good model for studying germ cells and freezing methods in other insects, as it is highly reproductive and easy to handle in laboratories.

In this study, we isolated the reproductive organs and germ cells from adult mealworms, developed an efficient method for isolating ovarioles from female adult mealworms, and established a cryopreservation protocol. This study can help develop the insect industry in the future by securing excellent insect species or contributing to preserving the genetic resources of rare and endangered insects.

## 2. Materials and Methods

### 2.1. Mealworm Rearing and Diet

Mealworm larvae were purchased from the market (Big Bit Mealworm Inc., Incheon, Republic of Korea) and bred at room temperature in a cycle of 14 L (Light):10 D (Dark). One hundred mealworms were bred in a plastic tray (32.5 × 20.5 × 23 cm). Bran was the major feed, and this was spread on the bottom 3–4 cm deep. Water was supplied by feeding carrots every 2–3 days.

### 2.2. Styrofoam-Feeding Tests

Fifty mealworm larvae were bred in a 90 mm petri dish with an average of 500 mg of Styrofoam for 20 days to check whether they have the ability to break it down. The Styrofoam weight was measured after 20 days.

### 2.3. Isolation of Testes, Ovaries and Germ Cells from Male and Female Adult Mealworms

The male or female adult mealworm was placed in a 1.5 ml tube and anesthetized with 70% alcohol. Then, it was placed on nonwoven fabric, and the head, carapace, wings, and upper part of the body were cut off with a scalpel. The testes or ovaries were isolated from the lower part of body and placed in a 35 mm petri dish with 2 mL Dulbecco’s phosphate buffered saline (DPBS) using forceps and a 1 mL syringe. The testes or ovaries were anatomically observed under a microscope as well as the sperms and ovarioles.

### 2.4. Histology of Testes and Ovaries from Male or Female Adult Mealworms

The testes or ovaries from the male or female adult mealworms were fixed by 4% paraformaldehyde for 24 h and immersed in 20% sucrose in PBS solution for 12 h. An optimal cutting temperature (OCT) compound (Miles Inc., Elkhart, IN, USA) was used to mount them; then, the samples were frozen at −56 °C. A 20 µm thick tissue section was cut using a Cryostat (Leica, Nussloch, Germany), and stained with DAPI.

### 2.5. Comparison of Ovary Viability of the Female Adult Mealworm between Slow-Freezing and Vitrificaiton

For slow-freezing, 2–3 ovaries of the female adult mealworm were placed in a cryovial with Dulbecco’s Modified Eagle Medium (DMEM) including 10% fetal bovine serum (FBS) and 10% dimethyl sulfoxide (DMSO), and frozen at −80 °C in the freezer, overnight. The vials were stored for 1 week in LN2 at −196 °C, and then the frozen ovaries were thawed in a water bath at 37 °C. For vitrification, 2–3 ovaries were treated for 5 min in pre-equilibrium solution composed of 7.5% dimethyl sulfoxide (DMSO) and 7.5 ethylene glycol (EG) supplemented with 10% FBS (DMEM). They were then immersed into a vitrification solution consisting of DMEM supplemented with 10% FBS, 15% DMSO and 15% EG for 1 min, and were then vitrified on a copper electron microscope (EM) grid into liquid nitrogen for at least 3 min. Afterward, the EM grids were sequentially transferred to 1.0, 0.5, 0.25, 0.125, and 0 M sucrose in DMEM at 37 °C to thaw the samples. Thawed ovaries of the adult female mealworm, after slow-freezing and vitrification, were fixed with 10% formalin (Sigma-Aldrich, St. Louis, MO, USA) for histology. Fixed tissues were washed, dehydrated, and embedded in Paraplast (Merck KgaA, Darmstadt, Germany). Paraffin-embedded tissues were sectioned using a microtome (Leica, Bensheim, Germany), stained with hematoxylin and eosin (H&E) (Sigma-Aldrich, St. Louis, MO, USA), and observed via light microscopy.

### 2.6. Ovarioles Isolation from Ovaries of Adult Female Mealworm Using Enzymatic and Mechanical Methods

For the mechanical method, the ovaries of the female adult mealworm were placed in a 35 mm culture dish with 2 mL DPBS, and then the ovaries were isolated mechanically using a 1 mL syringe by cutting the connecting node of the ovarioles. For the enzymatic method, the ovaries were left in 0.2% collagenase (DMEM) at room temperature (RT) for 20 min, and then the connecting node of the ovarioles was cut with a 1 mL syringe.

### 2.7. Vitrification and Viability of Alginate Encapsulated Ovarioles

To produce alginate-encapsulated ovarioles, purified sodium alginate (2%, *w*/*v*) was prepared by dissolving it in a 0.25 M aqueous D-mannitol solution. The ovarioles were put into alginate, and were further gelled in 50 mM CaCl_2_ for 2 min. After the removal of the CaCl_2_ with DPBS, the alginate-encapsulated ovarioles were placed on an EM grid, which were vitrified in the same way as mentioned above. Ovarioles without alginate capsules were used as a control. After thawing, they were stained with 5 μM calcein AM, 5 μM ethidium homodimer (Invitrogen, Waltham, MA, USA), and DAPI.

### 2.8. Statistical Analysis

A one way ANOVA in Statistical Analysis System (SAS) program was used for statistical analysis to determine the *p* value between various treatments. The differences were taken as significant when the *p* value was less than 0.05.

## 3. Results and Discussion

### 3.1. Changes in Weight of Mealworms after Feeding on Styrofoam

**Figure 1:**
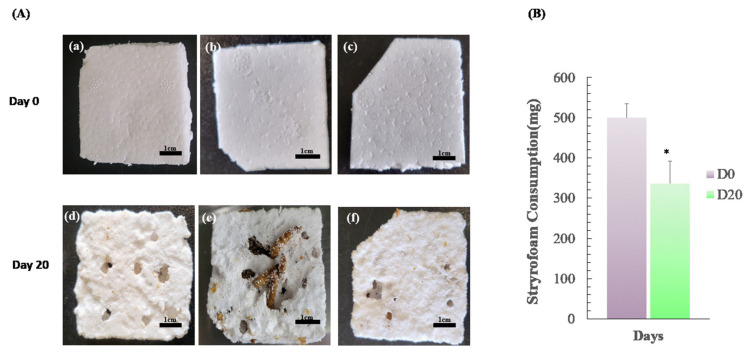
The ability of mealworms to break down Styrofoam. (**A**) Mealworms show changes before and after eating them Styrofoam. In a group of 50 mealworms, only Styrofoam was provided as feed for 20 days (a–c), and the mealworms were observed to decompose it (d–f). (**B**) After 20 days, the mealworms were isolated and the weight of the Styrofoam was compared with the first day (Day 0). All data are expressed as means ± SEM for triplicate measurements. The significant differences are indicated by asterisks (* *p* < 0.05).

Three groups of 50 mealworms were each fed 460 mg, 520 mg, and 520 mg of Styrofoam, respectively, and bred for 20 days. The quantity was eventually reduced to 340 mg, 280 mg, and 390 mg of Styrofoam, respectively. Although the Styrofoam intake was different for each group of mealworms, they ate the Styrofoam. The average Styrofoam consumption of 500 mg significantly dropped to an average of 336 mg (*p* < 0.05) after 20 days (Figure 1). It is thought that there are individual differences between mealworms, as each group has a different ability to decompose the Styrofoam. These results suggest that microorganisms in the mealworm gut can decompose Styrofoam [8]. Therefore, mealworms are considered an alternative to prevent environmental pollution by decomposing plastics [9]. The experiment confirmed that the mealworm could decompose Styrofoam. Cryopreservation studies of reproductive organs and germ cells are necessary to select mealworms with excellent Styrofoam-degrading traits and to preserve them from generation to generation.

### 3.2. Identification of Reproductive Organs and Germ Cells of Male and Female Adult Brown Mealworms (aBMs)

**Figure 2:**
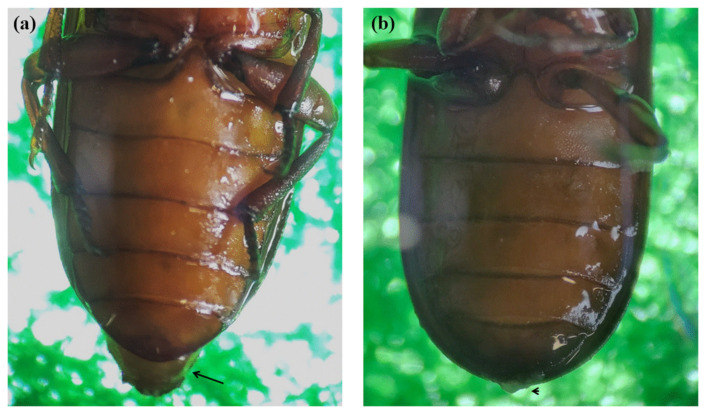
Distinguishing between female and male adult mealworms. Sexual difference of female adult brown mealworms is determined by the presence or absence of a protrusion on the fifth segment of the abdomen. Females ((**a**); arrow) have a sharper protrusion than males ((**b**); arrow-head).

**Figure 3:**
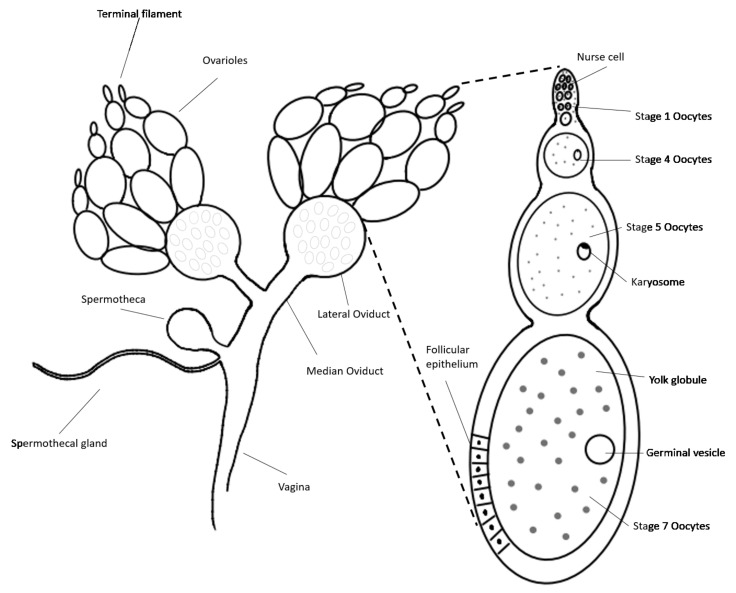
Schematic diagram of ovary structure and oogenesis in the female adult mealworm. The ovary consists of ovarioles, i.e., the basic unit of egg production. The yolk globule is inside the ovarioles, and the follicular epithelium surrounds them on the outside. Small ovarioles gradually develop and become mature follicles; eggs eventually ovulate from the mature ovariole.

Morphologically, male and female adult mealworms are distinguished according to the presence or absence of a protrusion at the tip of the fifth segment from the abdomen (Figure 2). An anatomical schematic diagram of the ovary of a female aBM was drawn in detail (Figure 3), as the germ cell development process in the ovary is much more complicated than in the testis. The ovary and female germ cells were isolated and frozen afterward. In the male, two testes produce the sperm, whereas the ovary consists of ovarioles with multiple stages of development; the ovariole is the basic unit of egg production. The yolk globule is present on the inside of ovarioles, where the follicular epithelium surrounds them. Small ovarioles gradually develop and become mature follicles; eggs ovulate from the mature ovarioles (Figure 3).

**Figure 4:**
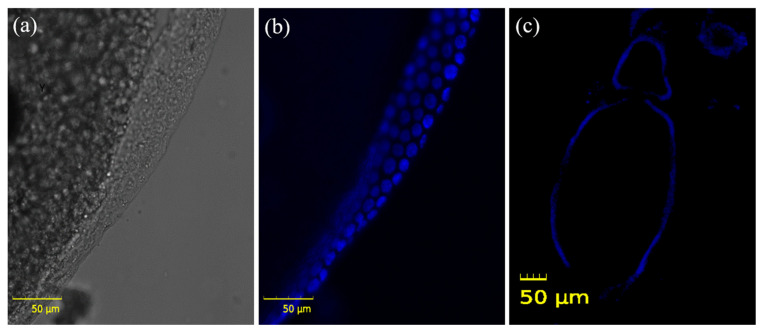
Identification of ovarioles from a female adult mealworm using frozen sections. (**a**) The yolk globule is in the ovariole, and (**b**) is surrounded by follicular epithelium. (**c**) The structure of the ovarioles was confirmed through DAPI staining.

**Figure 5:**
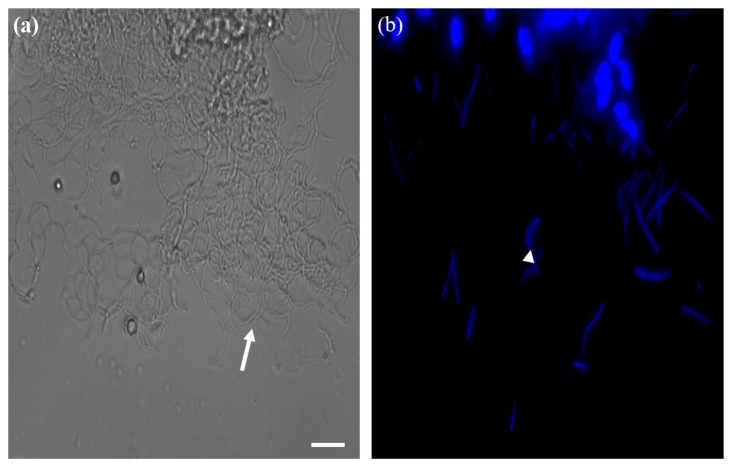
Histology of testis of male adult mealworm. (**a**) Sperms with long-tails (arrow) were observed in the testis and, (**b**) sperm nuclei (arrow-head) were stained with DAPI. Scale bars = 50 μm.

**Figure 6:**
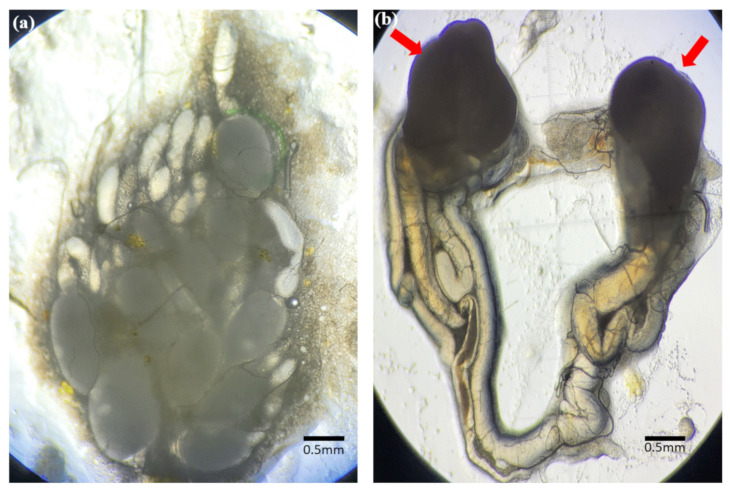
Reproductive organs isolated from female and male adult mealworms. (**a**) Various ovarioles at different developmental stages were observed in the female adult mealworm, and (**b**) two testes (red arrow) in the male adult mealworm.

Frozen sections were performed to confirm that the recovered reproductive organs were actual testes and ovaries, and the cell nuclei were stained with DAPI. In the ovary, yolks were confirmed inside the ovarioles, and the follicular epithelial cells around them were stained with DAPI (Figure 4). The long tails and heads of sperms stained with DAPI were identified in the testes (Figure 5). These findings proved that the ovaries and testes isolated from aBMs are real reproductive organs, including the ovarioles and sperms (Figure 6). This may provide basic data for reproductive research, as few studies have been conducted on aBM reproduction.

### 3.3. Comparison between Slow-Freezing and Vitrification for Adult Brown Mealworms (aBMs)

**Figure 7:**
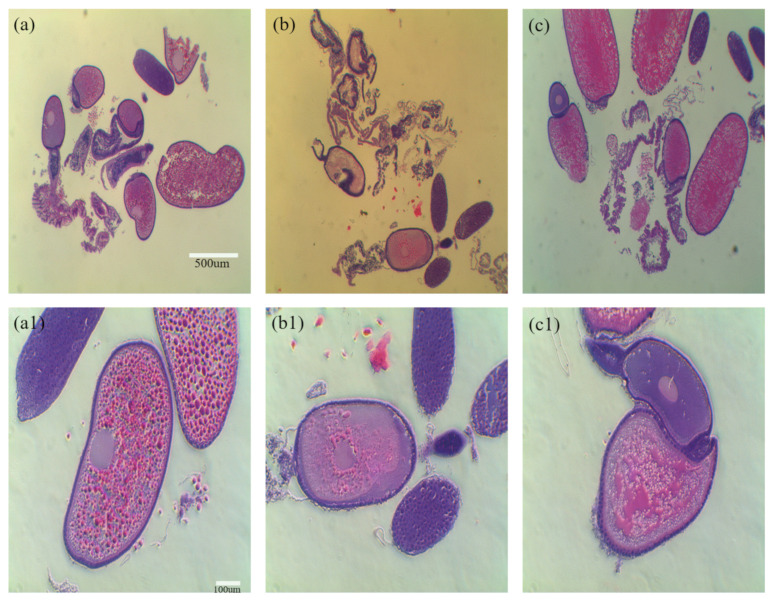
Comparison of the freezing methods used for ovaries isolated from female adult mealworm: slow-freezing and vitrification. The yolk of the ovariole was evenly distributed in the ovarioles in the control (**a**,**a1**). The yolk was more damaged in the slow-freezing group (**b**,**b1**) than in the vitrification group (**c**,**c1**), as the yolk of the ovarioles in the slow-freezing group is concentrated in a specific part. Scale bars: 500 µm (**a**–**c**), 100 µm (**a1**–**c1**).

The ovaries of female aBMs were frozen using slow-freezing and vitrification to determine which method was effective for freezing. After freezing using each method, the ovarian status of the tissue sections was confirmed during slow-freezing and vitrification. In the control group, the yolk was evenly spread in the ovarioles. However, the yolk was more damaged in the slow-freezing group than in the vitrification group (Figure 7). The cryopreservation of reproductive organs is used to preserve endangered species or species with superior genetics. There are two conventional methods of freezing: slow-freezing and vitrification. Although studies have been conducted on mammals [10,11], amphibians [12,13], and reptiles [14,15], few studies have been conducted on freezing the reproductive organs in brown mealworms. Although vitrification induces metabolic and osmotic damage to cells by using a high concentration of cryoprotectants (CPA), such as DMSO (up to ~8 M) [16,17,18], it improves cell viability and prevents intracellular ice formation (IIF). However, in slow-freezing, a low concentration of CPA (less than ~2 M) is used to reduce cell toxicity, thereby inducing IIF during freezing and thawing [19]. Therefore, slow-freezing can lead to cell damage. As mentioned above, the ovaries after slow-freezing might be more damaged than after vitrification, as it induces more ice crystals in the ovary.

### 3.4. Comparison of Enzymatic and Mechanical Methods for Ovarioles Isolation from the Ovaries of Female Adult Brown Mealworms (aBMs)

**Figure 8:**
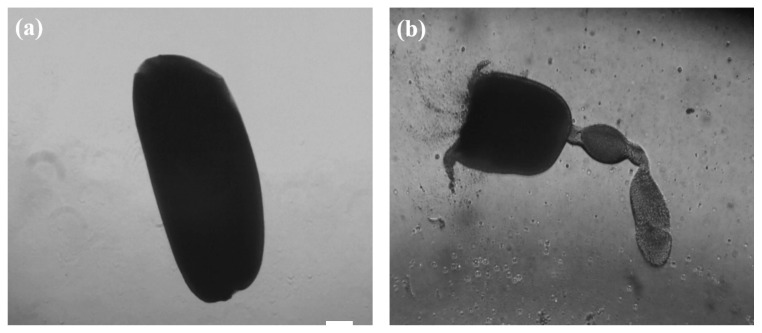
Comparison between mechanical and enzymatic methods used for ovarioles retrieval from female adult mealworms. (**a**) The mechanical method of isolating the ovarioles using a syringe maintains their integrity. (**b**) The enzymatic method using collagenase I damaged the outer membrane of the ovarioles. Scale bar: 50 µm (**a**,**b**).

Ovarioles were isolated from the ovaries of female aBMs, using the mechanical method (syringe) and the enzymatic method (collagenase I). The outer membranes of the ovarioles isolated using the enzyme treatment were damaged and burst (Figure 8a), whereas the ovarioles isolated using the mechanical method remained intact (Figure 8b) [20]. As the ovaries of female aBMs are large, the focus was on freezing the ovariole, which is the smallest unit that produces eggs. To determine which methods can efficiently retrieve ovarioles, they were isolated from the ovaries using the collagenase I enzyme and a mechanical method (syringe). As the outer layers of the ovarioles retrieved via the enzymatic method were severely damaged, the ovarioles were isolated using the mechanical method for freezing.

### 3.5. Effects of Vitrification on the Viability of Alginate-Encapsulated Ovarioles

**Figure 9:**
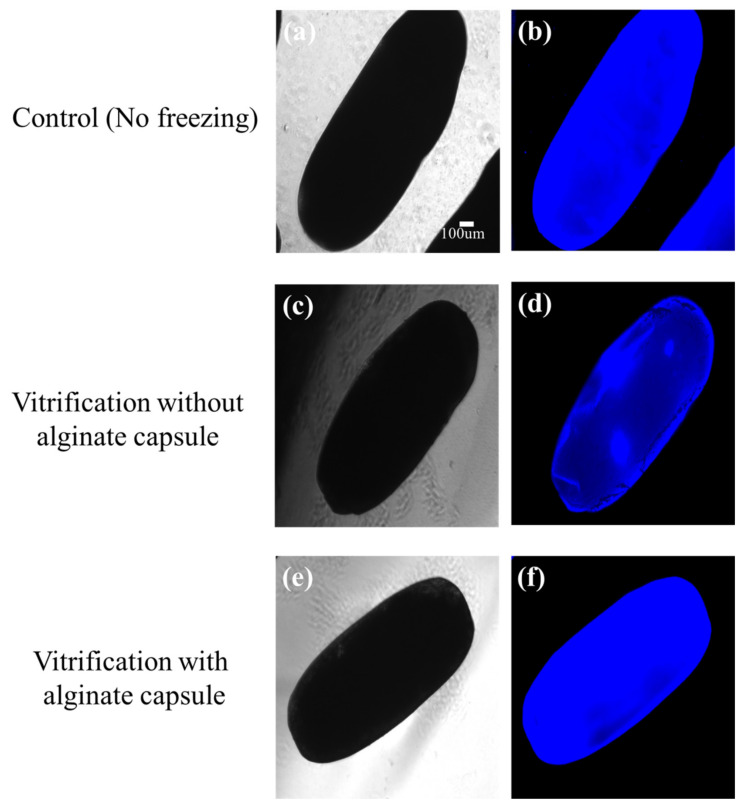
Viability of female adult mealworms by vitrification of the ovariole with or without an alginate-capsule. Alginate-encapsulated ovarioles were stained evenly, similarly to the control (**a**,**b**); the ovarioles without an alginate capsule (WOAC) had DAPI staining in a specific area (**c**,**d**); alginate-encapsulated ovarioles were ruptured less than the ovarioles WOAC (**e**,**f**). Scale bars = 50 μm.

When the ovarioles, with or without alginate microcapsules, were rapidly vitrified on an electron microscope (EM) grid; after thawing, the alginate-encapsulated ovarioles were stained evenly, similarly to the control, and were observed to have ruptured less than the ovarioles without an alginate capsule (WOAC), which showed DAPI staining in a specific area (Table 1, Figure 9). Alginate derived from brown algae is a biomaterial that is convenient to use in vitro because it becomes gelled at room temperature when it comes into contact with CaCl2. Therefore, alginate capsules have been used in 3D cell culture or cell transplantation in vivo, due to their biocompatibility, and because they experience less immune rejection [21,22]. In addition, when freezing alginate-encapsulated cells, the alginate capsule has been shown to protect the cells, as it prevents IIF in the cells during the thawing process [23]. Therefore, this is the same reason the survival rate of the alginate encapsulated ovarioles was higher than that without alginate-encapsulated ovarioles.

## 4. Conclusions

To preserve the excellent genetic characteristics of mealworms—a useful insect that can decompose Styrofoam, which is a cause of environmental pollution—we isolated reproductive organs, such as the ovaries and testes, and confirmed the presence of germ cells. First, a study on freezing the reproductive organs of the ovaries using slow-freezing and vitrification was attempted in brown mealworms, and a method for efficiently isolating the ovarioles from the ovaries was developed. It was demonstrated that the mechanical method of isolating the ovarioles was superior to the enzymatic method. The newly developed vitrification protocol using alginate capsules improved the ovarioles after cryopreservation. Therefore, this study contributes to the biobanking and fertility preservation of superior mealworm germ cells and endangered insects.

## Figures and Tables

**Table 1:** Comparison of the ovarioles’ viability between vitrification without or with an alginate capsule.

Group	No. of Total Ovarioles (%)	No. of Ruptured Ovarioles (%)	No. of Retrieved Ovarioles (%)
Vitrification without alginate capsule	13 (100)	7 (53.8)	6 (46.2)
Vitrification with alginate capsule	24 (100)	11 (45.8)	13 (54.2)

## Data Availability

The data presented in this study are available on request from the corresponding author.
